# Identification of dynamic models of microbial communities: A workflow addressing identifiability and modeling pitfalls

**DOI:** 10.1371/journal.pcbi.1013204

**Published:** 2025-12-01

**Authors:** Ana Paredes-Vázquez, Eva Balsa-Canto, Julio R. Banga

**Affiliations:** 1 Computational Biology Lab, MBG-CSIC (Spanish National Research Council), Pontevedra, Galicia, Spain; 2 Applied Mathematics Dept., Universidade Santiago de Compostela, Santiago de Compostela, Galicia, Spain; 3 Biosystems and Bioprocess Engineering, IIM-CSIC (Spanish National Research Council), Vigo, Galicia, Spain; Pázmány Péter Catholic University: Pazmany Peter Katolikus Egyetem, HUNGARY

## Abstract

Microbial communities, complex ecological networks crucial for human and planetary health, remain poorly understood in terms of the quantitative principles governing their composition, assembly, and function. Dynamic modeling using ordinary differential equations (ODEs) is a powerful framework for understanding and predicting microbiome behaviors. However, developing reliable ODE models is severely hampered by their nonlinear nature and the presence of significant challenges, particularly critical issues related to identifiability.

Here, we address the identification problem in dynamic microbial community models by proposing an integrated methodology to tackle key challenges. Focusing on nonlinear ODE-based models, we examine four critical pitfalls: identifiability issues (structural and practical), unstable dynamics (potentially leading to numerical blow-up), underfitting (convergence to suboptimal solutions), and overfitting (fitting noise rather than signal). These pitfalls yield unreliable parameter estimates, unrealistic model behavior, and poor generalization. Our study presents a comprehensive workflow incorporating structural and practical identifiability analysis, robust global optimization for calibration, stability checks, and rigorous predictive power assessment. The methodology’s effectiveness and versatility in mitigating these pitfalls are demonstrated through case studies of increasing complexity, paving the way for more reliable and mechanistically insightful models of microbial communities.

## Author summary

Microbial communities, vital for human and environmental health, are complex systems whose quantitative behaviors are not yet fully understood. Scientists employ mathematical models to study these communities, but developing accurate models of their dynamics is very challenging. Key difficulties include determining correct model parameters, ensuring model stability, and preventing the models from either under-learning from data or over-learning from noise. This paper presents a new, integrated methodology to overcome these obstacles. The approach provides a systematic workflow incorporating analyses of parameter identifiability, model stability, and predictive capabilities. By addressing these critical pitfalls, this research aims to facilitate the creation of more reliable and mechanistically insightful models, ultimately enhancing our understanding of microbial community dynamics.

## Introduction

A microbial community, often referred to as a microbiome, is a group of microorganisms that live together in a specific environment [1]. These microorganisms can include bacteria, archaea, fungi, and viruses. They interact with each other in complex ways, forming intricate ecosystems. These communities play crucial roles in various processes. For instance, the gut microbiota influences digestion and immunity, while the soil microbiome is essential for nutrient cycling and plant growth. Understanding microbial communities is vital for fields such as bio-medicine, microbiology, ecology, biotechnology, and agriculture, as they can impact human health and environmental sustainability [2].

Characterizing the nature of interactions within these systems helps reveal the roles of microbial species. Various qualitative and quantitative methods have been developed to analyze microbial community functions [3]. Quantitative approaches based on mathematical modeling are especially helpful. They provide valuable insights into the functioning of microbial communities, helping researchers to understand, predict, and potentially manipulate these complex systems [4–6]. The abstraction capabilities of these mathematical models are crucial for capturing underlying phenomena and linking the various scales at which these systems operate [7]. In this context, a number of network inference methods have been applied to characterize microbial interactions as static network models, sometimes mapping the inferred interactions with ecological motifs, such as cooperation, competition, commensalism, predation, and amensalism [8]. However, these network models offer a static view of microbial communities, capturing their status at a specific moment. In order to study changes over time, and time-dependent phenomena such as stability, response to disturbances, and succession, dynamic models are necessary.

Dynamic modeling is a particularly powerful framework that helps infer directionality and causality, predict time-dependent properties, and capture the dynamic behaviors of microbiomes in changing environments [9]. It can identify key microbes, molecules, and genetic determinants with significant causal effects on microbial community behaviors, predict their responses to perturbations, and guide the design of precise interventions to modify community functions [10]. Many existing frameworks for modeling the dynamics of microbial communities have been inspired by ecosystem modeling approaches, and include Lotka–Volterra, consumer–resource, trait-based, individual-based and genome-scale metabolic models, each one with its own scope, advantages and limitations [5,11,12]. In particular, Generalized Lotka-Volterra (GLV) and consumer-resource (CR) models are widely used [5,13], although their suitability depends on the origin of the available experimental data and the theoretical assumptions [14].

While various dynamic modeling paradigms exist, including agent-based models (ABMs) and partial differential equations (PDEs) for spatially explicit scenarios [15,16], ordinary differential equations (ODEs) are frequently used when spatial effects are averaged or not the primary focus [7,17]. ODEs effectively capture time-dependent processes like the complex interactions between microbial species, such as competition, cooperation, and predation. Moreover, they can incorporate the influence of environmental factors (e.g., nutrient availability, temperature, and pH) on microbial growth and interactions, and can be used to make quantitative predictions about the behavior of microbial communities under different conditions. These ODE-based models can help understand the underlying mechanisms driving community dynamics and design time-dependent interventions to manipulate these communities. In the remainder, we consider dynamic modeling of microbial communities using ODE-based models. In any case, it should be noted that several of the methods described below can be extended to handle PDEs, and probably ABMs.

Building these ODE dynamic models is challenging due to several factors. To begin with, microbial communities involve complex networks of interactions among microbes and with their environment. These interactions can be direct (e.g., competition, predation), indirect (e.g., through metabolite exchange), or higher-order (beyond simple pairwise interactions). Capturing all these interactions accurately and mapping them to the structure of an ODE-based model is difficult [4,5]. But, more importantly, even when the model structure is adequate, we still need to solve the identification problem, i.e., fitting the model to data and evaluating its predictive power.

The identification of dynamic nonlinear ODE-based models presents a complex set of challenges that stem from both the intrinsic properties of the models and the nature of available data. These challenges can significantly impact the accuracy, reliability, and predictive power of the resulting models. One of the primary challenges is the potential lack of identifiability. Identifiability refers to whether the model’s parameters can be uniquely determined from the available data [18–20]. If a model is not identifiable, different parameter values can produce the same output, making it impossible to know which ones truly reflect the underlying biology.

Given the model (as a set of ODEs) and the mathematical formulation of the measured quantities (e.g., microbial abundances, nutrient concentrations, etc.), lack of identifiability can manifest in two forms [21]:

- Structural Identifiability: this arises from the structure of the model and its mapping with the measured variables. Some parameters or combinations of parameters may be inherently unidentifiable, regardless of the quality or quantity of data available.
- Practical Identifiability: even when a model is structurally identifiable, limitations in the available data (such as noise, sparsity, or limited range) can render certain parameters practically unidentifiable.

A second challenge is related to the nonlinear nature of these models, which introduces additional layers of complexity to the parameter estimation process, notably the non-convexity of the optimization problem used in the fitting process. Also, it should be noted that the solutions of some nonlinear ODEs may not exist for all integration times. A phenomenon known as finite-time blow-up, also called explosive instability, can occur, where solutions approach infinity in a finite amount of time, which is often biologically unrealistic but mathematically possible in some models. This behavior has been observed even in relatively simple ecological models [22,23].

In this study, we address the identification problem within microbial communities and propose an integrated methodology to effectively tackle these challenges. Specifically, we examine four critical pitfalls that commonly undermine the identification of nonlinear ODE-based models:

- Identifiability issues, structural or practical: as mentioned above, can lead to unreliable parameter estimates or multiple equivalent solutions.
- Blow-up: if the model exhibits finite-time blow-up for certain parameter combinations, and the optimization algorithm explores these regions, this can lead to numerical issues that hinder optimization.
- Underfitting: this occurs when the estimation algorithm converges to a local optimum rather than the global optimum. It results in a model that fails to capture the full complexity of the underlying system dynamics.
- Overfitting: in this case, the model fits the noise in the data rather than the true underlying signal. Overfitted models often perform well on training data but fail to generalize to new scenarios, i.e., they lack predictive power.

These four issues are often intertwined and can exacerbate each other. For example, fitting a model with unidentifiable parameters might be more prone to overfitting and getting trapped in local optima. Blow-up dynamics can further complicate the optimization landscape and make it harder to find the true parameters. While these challenges are not unique to models of microbial communities, in our experience they arise more frequently and with greater severity within this class of problems, as further detailed in the Methods section below.

We demonstrate that if these pitfalls are not properly detected and mitigated, they can lead to significant modeling artifacts. These artifacts may include biased or inconsistent parameter estimates, unrealistic model behavior in certain regimes, poor generalization to new data or scenarios, and misleading interpretations of system dynamics. Ultimately, models suffering from these issues lack robust predictive power and may lead to incorrect conclusions about the system being studied.

To the best of our knowledge, these issues have not been thoroughly examined in the microbial communities literature. Only recently have a few studies begun to explore identifiability analysis for some of these models. Two of these works address structural identifiability specifically [24,25], while another considers both structural and practical identifiability [26]. Other critical challenges, however, remain largely unexamined.

Here, we present an integrated and systematic methodology to detect and address these issues simultaneously. This paper is structured as follows: first, we describe a comprehensive workflow to properly evaluate identifiability, perform robust model calibration, and assess the predictive power of the resulting fitted model. We also introduce a software implementation of this workflow that makes use of electronic notebooks, making it more accessible and user-friendly. Next, we apply our methodology to case studies of increasing complexity, including several examples of widely used canonical models, to demonstrate its effectiveness and versatility. Finally, we discuss the main findings, emphasizing the effectiveness of the proposed methodology in addressing the identified pitfalls.

## Methods

### Overview

We present a methodology for the robust calibration and validation of dynamic models of microbial communities described by nonlinear Ordinary Differential Equations (ODEs). Dynamic models of microbial communities, such as generalized Lotka-Volterra (gLV) or consumer-resource models, possess distinctive features compared to many other models in computational systems biology [28–30]. While challenges like nonlinearity and high dimensionality are common in systems biology, their specific combination and context in microbial ecology, plus the presence of other specific challenges, create a uniquely difficult parameter estimation problem that motivates a tailored computational workflow. The primary challenges are:

**Strong Nonlinearity and Highly Nonconvex Landscapes.** Microbial community models explicitly encode complex interaction networks through nonlinear terms. This inherent nonlinearity gives rise to complex, multimodal dynamics and, consequently, a highly nonconvex objective function landscape. This makes optimization susceptible to local optima, hindering the identification of globally optimal solutions.
**Dense Model Structures and Data-Driven Identifiability Issues.** Unlike many systems biology models with defined topologies (e.g. biochemical pathways), certain types of models like gLV often assume a dense, all-to-all interaction structure. This leads to a quadratic ($𝒪(N^{2})$) increase in parameters with the number of species (*N*), resulting in extremely high-dimensional systems. This structural complexity is compounded by severe identifiability challenges rooted in the symmetries of the interactions and the lack of sufficient informative data (given the large number of parameters). Many parameters, especially interaction coefficients, can be structurally or practically non-identifiable from typical community-level data. Data often consist of relative abundances or aggregated signals, which complicates the unique resolution of absolute parameter values. Observability is limited since some species or resources may be unmeasured or measured only coarsely.
**Phenomenological Nature and Proneness to Overfitting.** Compared to more mechanistic frameworks (e.g., metabolic models constrained by stoichiometry), gLV and similar models are largely phenomenological. The large number of parameters often lacks strong theoretical or prior constraints. This high degree of freedom, combined with what is often sparse time-series data, creates a significant risk of overfitting, where the model captures noise in the training data but fails to generalize.
**Potential for Numerical Instability.** Due to their nonlinear structure, these models (particularly gLV formulations) can exhibit solutions with finite-time blow-up (unbounded growth). This causes numerical instability during parameter estimation, as many candidate parameter sets lead to ODE solver failures. Calibration algorithms must therefore incorporate strategies to handle or constrain the search to dynamically stable regions.

Overall, although these distinctive characteristics and challenges are not strictly exclusive to microbial community models, the confluence of a dense parameter structure, the potential for overfitting and numerical instability, and a lack of mechanistic constraints does present a distinct and severe challenge. To the best of our knowledge, the literature lacks a comprehensive framework that systematically addresses this specific combination of issues, which is the gap our work aims to fill. As previously indicated, only recently have a few studies [24–26] begun addressing some of these issues (e.g., identifiability analysis for selected models). Thus, a key contribution of our paper is to introduce and demonstrate the latest methods for identifiability analysis and robust model calibration specifically in the context of microbial community modeling, where these challenges are particularly pronounced.

Our integrated computational workflow has been developed to address these issues and consists of three sequential phases, each subdivided into modular subtasks:

- Phase 1, *pre-estimation preparation*, begins with structural identifiability analysis (SIA) to determine whether model parameters are theoretically distinguishable. If simplification is required, users can remove non-identifiable parameters, reparameterize equations to reduce complexity, or explore alternative input-output mappings by adjusting perturbed or measured variables.
- Phase 2, *robust parameter estimation*, employs global optimization techniques to avoid local minima, paired with efficient adaptive ODE solvers and mechanisms to address numerical instabilities and overfitting. After estimation, practical identifiability analysis (PIA) is used to assess whether model parameters can be reliably estimated from the available data, considering measurement noise and experimental constraints. When needed, it can be followed by dynamic stability analysis via Jacobian eigenvalue evaluations to assess system robustness.
- Phase 3, *predictive power analysis*, checks possible over-fitting and its consequences. It also evaluates predictive power and generalizability by comparing simulations against experimental data and additional cross-validation tests with held-out datasets. The results can help users to refine the model if needed. Refinement options include simplifying the model structure, redesigning experiments to improve data quality, or collecting additional data to resolve ambiguities.

**Fig 1:**
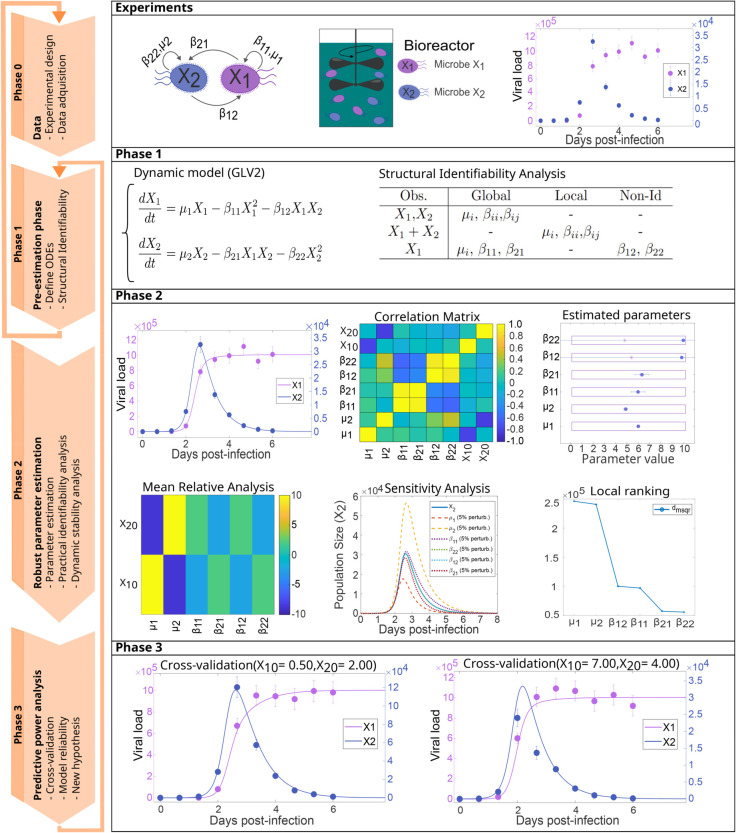
Diagram illustrating the workflow for model calibration. The figures correspond to a simple Generalized Lotka-Volterra model with 2 species in a competition scenario, specifically representing an *in-vivo* competitive mixture experiment with influenza strains in ferrets [27]. Further details about each phase of the workflow can be found in Methods.

This workflow, implemented via version-controlled scripts and interactive electronic notebooks, ensures reproducibility and transparency. By integrating theoretical rigor (e.g., identifiability checks, stability analysis) with computational robustness (e.g., global optimization, regularization), this pipeline minimizes risks such as over-fitting, non-identifiability, and unstable dynamics, yielding reliable models for real-world applications. The methodology is outlined in Fig 1. We describe the different phases in the following sections, with further technical details in S1 Text (Section 3).

### Pre-estimation preparation

The microbial communities under consideration are described by sets of non-linear Ordinary Differential Equations (ODEs) and the observation function as follows:

$$𝐟(\dot{𝐱},𝐱,𝐮,\theta ,t)=0$$ (1)

𝐲=𝐠(𝐱,t) (2)

where $\dot{𝐱}=\frac{d𝐱}{dt}$, $x(t)\in X\subset {\mathbb{R}}^{n_{x}}$ is the vector of state variables at time *t*, with initial conditions $𝐱(t_{0})=x_{0}(\theta )$; $\theta \in \Theta \subset {\mathbb{R}}^{n_{\theta }}$ denotes the vector of model parameters within the feasible parameter space Θ, and $𝐮\in U\subset {\mathbb{R}}^{n_{u}}$ corresponds to the external factors or inputs. $y\in {\mathbb{R}}^{n_{o}}$ regards the vector of *n*_*o*_ observables. We will regard as fully observed (FO) systems those for which all the state variables are amenable to experimentation and partially observed (PO), otherwise.

Structural identifiability analysis (SIA) seeks to determine which model parameters can be uniquely estimated from a given perfect data set, i.e., continuous and noise-free. It should be noted that in reality we never have perfect, noise-free data and measurements over infinite time. The question is whether parameter estimation is even theoretically possible given the model structure in Eqns (1)-(2). In general, we can distinguish:

- Global structural identifiability: parameters can be uniquely determined from the observations.
- Local structural identifiability: there is a finite set of parameter values that yield the same observations. In synthetic problems, parameters can be uniquely inferred near their nominal values, but multiple equivalent solutions may exist in the parameter space.
- Structural non-identifiability: there is an infinite set of parameter values that yield the same observations.

Structural identifiability is crucial for meaningful parameter estimation, as non-identifiable models yield unreliable and non-unique parameter values that may not reflect the true system properties. Inaccurate estimates of mechanistically significant parameters compromise the usefulness of the model to provide biological insights [21,31,32]. It also impacts model validation and interpretation, making it difficult to draw robust conclusions when parameters cannot be uniquely determined. Furthermore, identifying non-identifiable parameters informs new experiment design, guiding data collection and experimental strategies to improve parameter identifiability.

Several methods exist for studying SIA in nonlinear models [31,33], but they often rely on complex symbolic manipulations. While various software tools are available, they tend to be user-unfriendly and require significant expertise. Moreover, no single method applies universally to all models. These challenges create barriers that often lead to the neglect of this crucial step in model calibration.

After an initial screening based on our requirement to analyze structural global identifiability, as well as considerations of computational scalability, flexibility (including multi-experiment identifiability of both parameters and initial conditions), and previously reported performance [33], we selected the following tools for further evaluation:

- GenSSI2 [34] is a MATLAB-based tool that uses a generating series approach. It transforms the model equations into a system of polynomial equations on the parameters, analyzing their rank conditions to determine local identifiability and solving the system of equations to analyze global identifiability. However, its reliance on pure symbolic computations can make it computationally demanding for partially observed, highly non-linear systems.
- SIAN [35,36] is a Julia package that uses a randomized Monte-Carlo algorithm and an improved version of the Taylor series approach. It employs a differential algebra method that eliminates non-identifiable parameters through Gröbner basis computations.
- Structural Identifiability [37] is another Julia-based package that also uses differential algebra approach based on input-output equations. The method combines randomization and a differential elimination algorithm, and presents good scalability.

In principle, all these tools can handle both local and global identifiability, and can be applied to polynomial and rational ODE models. This selection offers a well-balanced combination of capabilities, performance, and suitability for the models under analysis. Each tool has unique strengths: GenSSI2 is effective for smaller to medium-sized models but can struggle with scalability due to its symbolic computation overhead, while SIAN and Structural Identifiability employ symbolic-numeric randomized algorithms and leverage Julia’s modern computational advantages for improved scalability and efficiency.

### Robust parameter estimation

Dynamic model calibration is the process of adjusting the parameters to ensure accurate representation of the biological system’s behavior based on available data [38]. This process is typically framed as an optimization problem aimed at minimizing discrepancies between model predictions and observed data. Here we adopt a single-shooting approach, where the initial value problem described by the ODEs is solved for each evaluation of the cost function [39]. Other options are discussed elsewhere [40,41].

The choice of objective function plays a crucial role in quantifying this mismatch, as it directly impacts the accuracy and reliability of the calibration. The objective function encapsulates characteristics of the measurement process and incorporates prior knowledge. In frequentist approaches, it typically takes the form of the maximum (log)-likelihood function (details in S1 Text, Section 3.1). When standard deviations are known, this function follows a weighted least-squares form:

$$J_{\text{lsq}}(\theta )=\sum_{e=1}^{n_{e}}\sum_{o=1}^{n_{o}^{e}}\sum_{s=1}^{n_{s}^{e,o}}{\left[\frac{{ym}_{s}^{e,o}-{𝐲}_{s}^{e,o}(\theta ,t_{s}^{e,o})}{\sigma_{s}^{e,o}}\right]}^{2}$$ (3)

Where *n*_*e*_, $n_{o}^{e}$ and $n_{s}^{e,o}$ regard the number of experiments, the number of observables in a given experiment *e* and the number of sampling times for the specific observables and experiment, respectively. ${ym}_{s}^{e,o}$ represents the measured data at a given sampling time *s* for a specific observable *o* in the experiment *e*; ${𝐲}_{s}^{e,o}(\theta ,t_{s}^{e,o})$ denotes the model prediction for the sampling time $t_{s}^{e,o}$; and $\sigma_{s}^{e,o}$ is the standard deviation of noise. The estimation problem is formulated as the minimization of one of these objective functions subject to differential and algebraic constraints (i.e., the ODEs describing the dynamics plus the observation function and bounds on the parameters, $\theta_{\text{min}}\leq \theta \leq \theta_{\text{max}}$).

In the above formulation, the parameter space is often high-dimensional and the problem is strongly non-convex, characterized by multiple local minima, flat regions, and possibly discontinuities caused by solver failures or finite-time blow-ups. These complexities make traditional gradient-based local optimization methods insufficient on their own, as they tend to get trapped in local minima and depend heavily on the initial guess.

To overcome these challenges, we adopt a global optimization strategy that can efficiently explore the parameter space and avoid premature convergence to suboptimal solutions. Specifically, we employ *enhanced Scatter Search* (eSS), a metaheuristic algorithm that integrates global search heuristics with periodic local refinement steps [42,43]. The key advantages of eSS include:

1. Balance of exploration and exploitation: eSS maintains a diverse population of candidate solutions to explore the parameter space broadly (exploration), while leveraging local optimization techniques to fine-tune promising solutions (exploitation). This balance helps in efficiently navigating complex landscapes typical of nonlinear ODE parameter estimation.
2. Robustness to nonconvexity and noise: the metaheuristic nature of eSS allows it to handle nonconvex and rugged objective functions, including those with discontinuities or noisy evaluations caused by numerical integration issues. This robustness is critical when the underlying ODE solver occasionally fails or produces invalid results (NaN, Inf).
3. Reliable convergence properties: compared to other popular stochastic methods such as genetic algorithms and other more recent evolutionary computation methods, eSS has demonstrated superior convergence speed and reliability in a range of parameter estimation benchmarks, due to its strategic combination of global and local search components [44,45]. Recent benchmarking studies have also illustrated its superiority over sophisticated multi-start gradient-based local methods [43].
4. Flexibility and adaptability: eSS is flexible enough to be combined with problem-specific constraints and can incorporate parallel evaluations, which is beneficial when solving computationally expensive ODE integrations [44,45].

Given these strengths, we found eSS particularly well-suited for the parameter estimation challenges typical of the class of problems considered here. It allows us to obtain high-quality parameter estimates with a higher probability of identifying global or near-global optima, ultimately improving the predictive accuracy and reliability of the fitted models, and justifiying its selection as the global optimization solver in our workflow.

#### Stability analysis.

In the above formulation, the estimation relies on repeatedly solving the ODE system for different parameter values within an optimization loop. If the ODE system is unstable (e.g., exhibits blow-up) for even a small region of parameter space, the simulations will become unreliable or impossible to complete. For certain parameter values, the numerical solver might fail, produce nonsensical results (NaN, Inf), or take excessively long to simulate. This disrupts the optimization process, making it difficult to evaluate the objective function and navigate the parameter space. Even when convergence to a good fit is achieved, it is important to check if the calibrated model is stable.

The global optimization eSS metaheuristic in our workflow, described in the previous section, explores the parameter space broadly and therefore is prone to encounter blow-ups. However, unlike multi-start local optimization methods, which often fail due to blow-up in large portions of the parameter space, this hybrid strategy can effectively handle cases where the ODE integration returns NaN or Inf. As illustrated below with several case studies, this method successfully identifies parameter sets that yield good data fits while avoiding blow-up over the time horizons of interest.

However, an important issue arises with some of the solutions found by this approach. While they fit the data well and exhibit no blow-up over the observed time interval, extending the simulation beyond this horizon reveals that certain trajectories eventually diverge. This indicates that these parameter sets correspond to models that are locally valid but globally unstable. Recognizing this limitation, we implemented a stability analysis to assess the long-term behavior of the fitted models.

Our strategy for stability analysis for these nonlinear dynamical systems begins by identifying equilibrium points and then describing the system’s behavior around these points. This is achieved by linearizing the system using the Jacobian matrix at each equilibrium point, which captures the local dynamics. The stability is then determined by analyzing the real parts of the eigenvalues of this Jacobian matrix. If all real parts are negative, the equilibrium is asymptotically stable; if at least one is positive, it is unstable; and if all are non-positive with some being zero, it is neutrally stable. Based on the patterns of these eigenvalues, equilibrium points can be further classified as stable or unstable nodes, saddle points, spirals, or centers. To understand the broader system behavior beyond local stability, numerical simulations are used to explore global dynamics, including periodic orbits or chaotic behavior. For complex systems, more advanced numerical methods can be employed to reveal aspects like basins of attraction, limit cycles, and chaotic regimes. This approach is applicable to a wide range of ODE systems and is described in more detail in S1 Text (Sections 3.5 and 7.6.5).

This procedure enables us to classify whether a given parameter set leads to a model that is not only a good fit over the data horizon but also dynamically stable in a broader sense. In doing so, we can filter out solutions that are prone to divergence beyond the observation window, thereby increasing confidence in the predictive power and robustness of the inferred models. This post-fit stability check is a critical addition when working with nonlinear ecological models, as it helps distinguish between transiently accurate but unstable solutions and those that reflect plausible long-term ecological dynamics.

#### Practical identifiability analysis (PIA).

To assess the practical identifiability of parameters, our workflow uses the Fisher Information Matrix (FIM), computing parameter confidence intervals derived from the Cramèr-Rao inequality, and examining the correlation matrix [19,20]. The process starts by constructing the FIM (eqn 4), which quantifies the amount of information the data is expected to provide about the parameters:

$$ℱ=\underset{{\text{y}}_{\text{m}}|\mu }{E}\left\{\left[\frac{\partial J_{lsq}(\theta )}{\partial (\theta )}\right]{\left[\frac{\partial J_{lsq}(\theta )}{\partial (\theta )}\right]}^{T}\right\}$$ (4)

where *E* represents expected values and μ a value of the parameters, hopefully close to their real value (see below).

Computing the FIM involves calculating sensitivity matrices, which represent how parameter changes affect the model output, and incorporating assumptions about the measurement noise. The local parametric sensitivities for a specific experiment (*e*), observable (*o*), and sampling time (*t*_*s*_) read as follows:

$$S_{\theta }^{e,o}(t_{s}^{e,o})=\frac{\partial {𝐲}^{e,o}}{\partial \theta }(t_{s}^{e,o});\;\theta =1,\ldots ,n_{\theta }$$ (5)

A key step is to evaluate the FIM’s properties: a singular or ill-conditioned FIM suggests practical non-identifiability, indicating that parameters or combinations of parameters cannot be reliably estimated from the data. The condition number and eigenvalues of the FIM can provide further insights into the degree of identifiability, with small eigenvalues or large condition numbers signaling potential issues.

Subsequently, confidence intervals for the parameters can be approximated using the Cramèr-Rao inequality, which states that the inverse of the FIM provides a lower bound on the variance of unbiased estimators:

$$𝒞\geq {ℱ}^{-1}$$ (6)

where 𝒞 is the covariance matrix. By taking the square root of the diagonal elements of the inverse FIM, one obtains estimates of the standard errors for each parameter, which are then used to construct confidence intervals. Wide confidence intervals indicate poor practical identifiability, meaning that even with the assumed data quality, the parameters cannot be estimated with high precision. These wide intervals suggest that different parameter values, spanning a considerable range, could plausibly explain the observed data, thus hindering accurate parameter determination.

In practice, one typically evaluates the observed Fisher information at a point estimate (e.g., the maximum likelihood or nonlinear least squares estimator) and uses its inverse as a local approximation to the estimator’s covariance [20]. Although knowledge of the true parameter is not required, the Cramér–Rao lower bound is defined in terms of the information at the true value. Therefore under standard regularity conditions and for sufficiently large samples, the information at the estimate is a consistent proxy for the true information. This approximation is most reliable when the estimate is close to the truth, the parameter lies in the interior of an identifiable and smooth model, and the log-likelihood is approximately quadratic near the optimum. By contrast, pronounced nonlinearity, multimodality, boundary constraints, or nearly flat likelihood directions can render FIM-based confidence intervals and correlation measures inaccurate. In those situations, alternatives such as bootstrapping are recommended [46].

Finally, the correlation matrix, derived from the inverse of the FIM, offers insights into the interdependencies between parameter estimates. Off-diagonal elements of this matrix reveal the correlation coefficients between pairs of parameters. High correlation values, close to 1 or -1, suggest that these parameters are not independently identifiable, i.e., changes in one parameter must be compensated by changes in the correlated parameter to maintain a similar model fit to the data. This correlation implies redundancy in the parameterization and can guide model simplification or experimental redesign to improve identifiability. By collectively analyzing the FIM’s condition, the Cramèr-Rao based confidence intervals, and the parameter correlation matrix, one can gain a comprehensive understanding of the practical identifiability of parameters in ODE models, highlighting parameters that are poorly identifiable and suggesting potential remedies such as model refinement or improved experimental design.

### Predictive power analysis

To assess the predictive power of a calibrated ODE model, a crucial next step is to investigate potential **overfitting**. Initially, this can be achieved by examining the residuals, which are the differences between the experimental data and the model predictions at the time points where data was collected. If the model is appropriately calibrated and not overfitting the training data, the residuals should exhibit a random distribution around zero, with no discernible patterns or trends. In a well-specified model with additive homoscedastic errors, in-sample residuals should behave like white noise, i.e. approximately zero-mean, uncorrelated, and of constant variance, with no systematic structure when plotted against time. In other words, assuming Gaussian errors in the data, residuals should also be approximately normal [20,47,48].

Visual inspection of residual plots, such as plotting residuals against time or predicted values, is helpful. Ideally, the residuals should resemble white noise, indicating that the model has captured the underlying signal and the remaining variation is attributable to random error rather than systematic model deficiencies. However, residual diagnostics alone do not reliably detect overfitting, which is better assessed via out-of-sample evaluation or cross-validation [20,48,49].

Thus, following residual analysis, a more robust evaluation of predictive power requires **cross-validation**. This involves partitioning the available data into training and validation sets. The model is then calibrated using only the training data to obtain parameter estimates. Subsequently, the predictive capability of the calibrated model is assessed by comparing its predictions against the validation data, which was not used during calibration. If possible, this process should be repeated multiple times for several sets of validation data. The predictive accuracy can be quantified using metrics like the root mean squared error (RMSE) or normalized root mean squared error (NRMSE) between the model predictions and the validation data across all folds. Consistently good predictive performance on the validation sets, comparable to the fit on the training data, indicates robust predictive power and absence of overfitting.

If the model demonstrates good predictive power and no signs of overfitting, the final step involves evaluating the **mechanistic plausibility** of the model fit. This is crucial, especially when dealing with models built upon mechanistic principles. The estimated parameter values should be examined for their magnitudes and signs in the context of their mechanistic interpretation. For instance, parameters representing rates of biological processes should have positive values and magnitudes that are biologically reasonable. Similarly, the signs of parameters governing interactions should align with the expected mechanistic relationships (e.g., a parameter representing inhibition should have a negative effect).

Mechanistic plausibility can go beyond general guidelines (i.e., verifying that parameter signs and magnitudes are consistent with expectations) to a domain-specific formulation that specifies the concrete criteria applicable to microbial community models. In this context, mechanistic plausibility checks can draw on established biochemical, microbiological and ecological principles [19,50–52]. For example, maximum specific growth rates (*μ*) should be positive and within realistic ranges (e.g., 0.1–$2\;h^{-1}$ for many bacteria), mortality or washout rates should be non-negative and generally smaller than growth rates, and interaction coefficients should have signs consistent with their ecological roles (positive for facilitation or cross-feeding, negative for competition or inhibition) and magnitudes that are biologically reasonable. Half-saturation constants (*K*_*s*_) should be positive and within typical affinity ranges for the substrate (*μ*M–mM), stoichiometric yields should not exceed theoretical metabolic limits, and parameter values should respect mass and energy balances. Moreover, feasible parameter sets should allow coexistence only under realistic nutrient inflow and removal rates, consistent with known microbial ecology constraints. Such domain-specific checks ensure that the model remains interpretable and biologically grounded even when statistical fit and predictive power are high.

Deviations from expected ranges or signs for mechanistically meaningful parameters can suggest issues with the model structure, identifiability problems, or inconsistencies between the model and the underlying mechanisms, even if the model exhibits good statistical fit and predictive power. This mechanistic evaluation provides a crucial layer of validation beyond purely statistical measures, ensuring that the model not only fits the data but also offers a plausible and interpretable representation of the system under study.

### Software implementation

We developed our workflow as a unified, Matlab-based pipeline that integrates the previously mentioned three tools for structural identifiability analysis (SIA) alongside estimation and practical identifiability analysis (PIA) methods using AMIGO2 [53]. AMIGO2 (*Advanced Model Identification using Global Optimization*) is a powerful toolbox for dynamic modeling and optimization, offering a broad selection of nonlinear optimization solvers. These include direct and indirect local methods, multi-start local approaches, global stochastic algorithms, and hybrid optimization techniques. Additionally, AMIGO2 facilitates PIA.

To enhance computational efficiency, particularly for demanding tasks such as parameter estimation, using AMIGO2 in this framework enables automatic generation of C-compiled code, significantly improving performance. Furthermore, we extended the pipeline with additional code to support other key steps, such as stability analysis. Several case studies, based on commonly used models of microbial communities, were implemented and tested within this workflow.

The resulting integrated software is available in both electronic notebook format (live scripts) and as standard MATLAB scripts. It features the following key components:

- **Pre-estimation analysis:** the workflow simplifies model definition, data integration, and identifiability analysis, ensuring a solid foundation for parameter estimation. This module efficiently handles multi-experiment datasets, incorporates data visualization tools, and facilitates structural identifiability analysis using several methods, so non-expert users can determine whether model parameters can be uniquely inferred.
- **Robust parameter estimation:** a diverse set of global and local optimization algorithms enhances the reliability and accuracy of parameter estimation. Sensitivity analysis, practical identifiability assessment, and stability analysis help refine parameter estimates. The framework also supports easy cross-validation with additional datasets and leverages parallel computing for improved efficiency and scalability.
- **Predictive power analysis:** to ensure the model produces reliable and meaningful predictions, the workflow includes sensitivity analysis, statistical goodness-of-fit tests and cross-validation to assess model performance. These methods enable model comparison, interactive plotting, time series visualization, and statistical summarization, aiding in result interpretation. Furthermore, automated report generation and documentation via electronic notebooks streamline reproducibility and facilitate effective communication of findings.

## Results

We provide here a summary of the key findings, with further details available in S1 Text (Section 7). We evaluated our integrated computational workflow using a set of canonical models representing common frameworks for modeling microbial communities (Table 1). These models range from Generalized Lotka-Volterra (gLV) systems of varying complexity to more intricate models involving resource competition, phage dynamics, and synthetic gene circuits. To rigorously test the workflow and assess its robustness against experimental variability, we generated synthetic pseudo-experimental datasets for each model, incorporating different types and levels of Gaussian noise. This use of synthetic data provides a ground truth for evaluating parameter recovery and model predictive capabilities.

**Table 1:** Overview of case studies. The first row shows abbreviated model names. *n*_*x*_ denotes the number of state variables, and $n_{\theta }$ the number of parameters. GLV denotes generalized Lotka-Volterra. For the **GLV2** model, two subcases were analyzed: competition and coexistence. Further details are provided in S1 Text (Section 7).

Feature	GLV2	GLV3	MGLV	CompV	PCM	EPHP
Reference	[27]	[24]	[54]	[55]	[56]	[57]
Description	GLV 2 species	GLV 3 species	GLV 12 species	Competition with virus	Phage cocktail	Enhanced protein production
Microorganisms	Influenza virus	Generic microbiome	Gut microbiome	Two bacteria and virus	Bacteria and phages	Synthetic *E. coli* community
*n*_*x*_	2	3	12	4	5	5
$n_{\theta }$	6	12	156	7	13	19

### Phase 1: Structural identifiability analysis

We initiated the workflow with structural identifiability analysis (SIA) using GenSSI2, SIAN, and Structural Identifiability. The analysis covered scenarios ranging from ideal (fully observed states, known initial conditions) to realistic (partially observed states, unknown initial conditions). As summarized in Table 2, all models were found to be structurally identifiable under full observation. However, limitations arose under partial observation; for instance, in the GLV2 case, measuring only total biomass or a single species abundance resulted in non-identifiability, consistent with [26] and highlighting how experimental constraints impact parameter determination.

**Table 2:** Summary of SIA results. FO: Fully Observed, PO: Partially Observed, GI: Globally Identifiable, NGI: Non-Globally Identifiable (i.e. only a subset of parameters are identifiable). Last column indicates which methods were successful. Key: 1 = GenSSI2, 2 = SIAN, 3 = Structural Identifiability. Full details, including the partially observed schemes considered, can be found in S1 Text (Sections 7.5.2, 7.6.2, 7.7.2, 7.8.2, 7.9.2 and 7.10.2).

Model	Measurements	SIA Result	Method
GLV2	FO	GI	1, 2, 3
	PO	NGI	2, 3
GLV3	FO	GI	1, 2, 3
	PO	NGI	2, 3
MGLV	FO	GI	3
CompV	FO	GI	2, 3
PCM	FO and PO	GI	2, 3

In generalized Lotka–Volterra (gLV) models, there are scenarios in which certain parameters are locally but not globally identifiable. This means that while the parameters can be uniquely determined in a neighborhood of the true values (i.e., locally), there may exist multiple distinct parameter sets that produce exactly the same model output, making it impossible to distinguish between them based solely on the data. In contrast, global identifiability implies that there is a unique parameter set (within the parameter space) consistent with the observed outputs. Understanding and resolving these ambiguities is crucial for reliable parameter estimation and model interpretation.

We note that the presence of parameters that are locally but not globally identifiable often reflects underlying symmetries in the model structure. In particular, permutation symmetries can give rise to multiple distinct parameter sets that produce indistinguishable outputs. However, in our analysis of generalized Lotka–Volterra (gLV) models, we found that when the system is fully observed (i.e., when all species are individually measured over time) the model parameters are globally identifiable under generic conditions. This suggests that the gLV model, in its standard form, does not inherently suffer from non-identifiability due to structural symmetries.

In contrast, the instances in which we observed only local identifiability (or complete non-identifiability) corresponded to partially observed scenarios, such as when only the combined abundance of two or more species is measured. In such cases, the permutation-like symmetries arise not from the model itself, but from the structure of the input–output mapping induced by the observational process. The resulting lack of global identifiability is therefore a consequence of limited or aggregate measurements.

To address these issues, we highlight two common strategies for restoring identifiability: (i) refining the experimental design to provide more informative or disaggregated measurements (e.g., distinguishing between individual species), and (ii) reformulating the model through reparameterization or by introducing biologically or physically motivated constraints that remove unidentifiable degrees of freedom.

The performance of SIA tools varied. GenSSI2’s symbolic approach was effective for simpler models but struggled with larger partially observed ones due to computational demands. SIAN and Structural Identifiability, combining symbolic and numerical techniques, handled more complex models efficiently. The EPHP model could not be analyzed due to its discontinuous formulation, a limitation of current SIA tools assuming continuous dynamics.

### Phase 2: Parameter estimation and practical identifiability

This phase addresses the challenges of finding reliable parameter estimates from noisy data, considering potential non-convexity and numerical issues.

#### Optimization strategies and performance.

**Fig 2:**
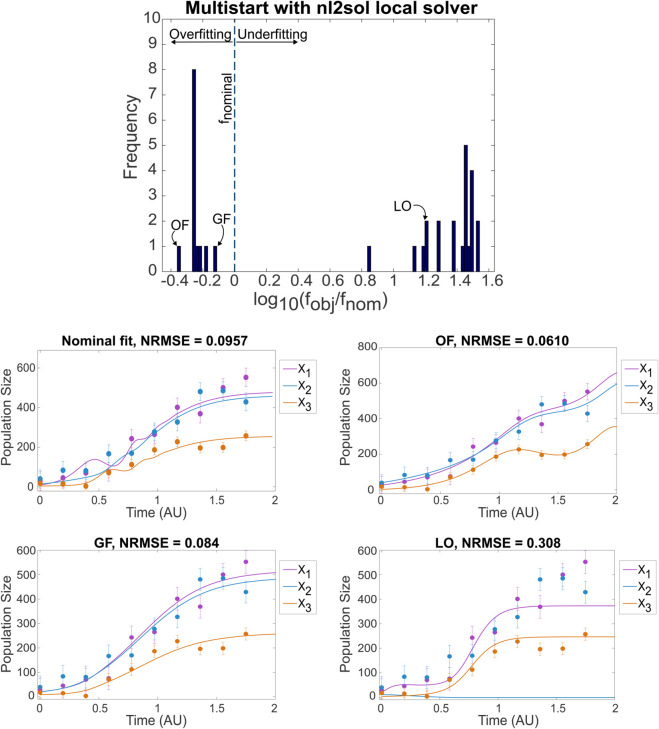
Case study *GLV3*: multistart optimization using the nl2sol local solver. Top figure shows a histogram of the 35 optima obtained in 200 runs, highlighting the presence of overfitting (OF), underfitting (local optimum, LO), and good fit (GF) solutions. The *x*-axis shows the $\log_{10}$ of the ratio between the objective function achieved and that of the nominal vector of parameters. Figures below present the nominal system behavior and show examples for the OF, GF, and LO cases, respectively.

We first explored multistart local optimization using least-squares optimizers (lsqnonlin, nl2sol) [53]. This revealed significant challenges across most models, particularly non-convexity (multiple local optima) and model instability (numerical blow-ups). For example, in the GLV3 case with 10% noise (Fig 2), only 17.5% of runs converged, and only 6% reached objective function values near the nominal (true) value. Many runs terminated due to blow-ups or converged to poor local optima (LO) or potentially overfitting solutions (OF).

Overall, multistart local methods showed acceptable performance only for the simplest cases (GLV2) and moderate effectiveness for PCM, achieving 70% good fits. For more complex cases, such as GLV3 and particularly MGLV models, convergence rates were significantly lower. Blow-ups occurred frequently (over 80%, even with tight bounds), and there was a high risk of converging to suboptimal solutions or overfitting. The high-dimensional MGLV model was especially challenging (good fits in only 12% or runs), often failing to accurately reproduce system dynamics, even when using noise-free data. In the CompV and EPHP cases, while most of the multistarts converged, good fits were scarce: only 13% of runs for EPHP and 30% for PCM achieved satisfactory results.

In contrast, the enhanced Scatter Search (eSS) global optimization algorithm exhibited significantly greater robustness. Convergence rates were consistently high (exceeding 95% overall and 85% for MGLV), effectively navigating complex optimization landscapes. While eSS did not completely eliminate challenges such as potential overfitting or local optima traps in highly complex models (e.g., MGLV, GLV3), it consistently delivered much more reliable results than multistart local methods. Additionally, the computational cost using eSS was very reasonable, ranging between 1 and 2 minutes for all cases on a standard PC with Intel i5-13500 CPU (further details available in S1 Text, Section 7.1).

#### Practical identifiability and mechanistic interpretation.

After optimization with eSS, we evaluated practical identifiability using the FIM to derive parameter correlations and confidence intervals (CIs), alongside sensitivity analysis. Results indicated a consistent decline in identifiability with increasing model complexity and noise. For simpler models like GLV2, parameters were reasonably well-defined with narrow CIs under full observation, although some correlations emerged (e.g., between $\beta_{12}$ and $\beta_{22}$, and between growth rates $\mu_{i}$ and initial conditions). Sensitivity analysis confirmed that parameters with larger CIs had less influence on system states (see Fig C and Fig E in S1 Text).

However, for more complex models (GLV3, CompV, PCM, MGLV), PIA revealed significant limitations. Even when structurally identifiable, parameters often had wide CIs and strong correlations, especially with noisy data. This indicates that, despite achieving a good fit, the available data may not be adequate for accurately estimating all parameters. In other words, the insufficient information content of the data rendered it inadequate for reliable model calibration.

Crucially, poor practical identifiability can compromise the mechanistic interpretation of the model. This issue was evident in GLV models where estimated interaction coefficients ($\beta_{ij}$) frequently had signs opposite to the ground truth values, even for models achieving a good fit (low RMSE/NRMSE). Such sign errors imply a misrepresentation of the underlying biological interactions (e.g., predicting competition instead of facilitation). Fig 4 illustrates this for the MGLV model, showing sign disagreements increasing with noise but present even in the noise-free case. This highlights inherent limitations in extracting mechanistic details when practical identifiability is poor, often linked to insufficient information content in the experimental data. Stability analysis was also performed on the best parameter sets to ensure they did not lead to unstable dynamics under the calibration conditions.

### Phase 3: Predictive power analysis

**Fig 3:**
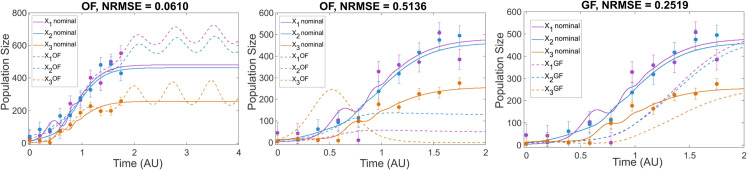
Case study *GLV3.* Left figure: An overfitted solution OF that agrees very well with the data but shows oscillatory behavior (and eventually blow-up) after *t* = 2.0. Center figure: cross-validation of the same OF for different initial conditions, showing very poor predictive value. Right figure: cross-validation of a good fit (GF), showing that due to practical identifiability issues, the agreement with the data is not very good, although ultimately predicts well steady state values at final time.

The final phase assessed model generalizability and overfitting risk using cross-validation with held-out data, also incorporating datasets from novel experimental conditions. Our analysis confirmed that overfitting is a significant risk across all models. Solutions achieving excellent fits to the training data often failed to predict system behavior accurately under new conditions. The GLV3 case study provides a clear example (Fig 3): an overfitted solution (OF) matches the training data well but exhibits unstable dynamics long-term (left panel) and performs poorly in cross-validation with different initial conditions (center panel). In contrast, a better-calibrated, though not perfectly identifiable, solution (GF) captures the trends under new conditions more reliably (right panel).

This underscores the insufficiency of relying solely on goodness-of-fit to training data and highlights the necessity of cross-validation for evaluating true predictive power. Predictive capability generally decreased with increasing model complexity and noise. While simpler models could maintain some predictive power if carefully calibrated, complex models like MGLV showed severely compromised prediction, even when calibrated on noise-free data, linking back to the practical identifiability limitations discussed in Phase 2.

Recognizing that obtaining additional experimental data for cross-validation can be challenging, we also explored several techniques for residual analysis and concluded that Quantile-Quantile (Q-Q) plots can be used as a potential indicator of overfitting based solely on training data (details in S1 Text, Section 7.6.4).

Overall, this phase emphasizes the critical need for rigorous validation beyond the initial calibration dataset to ensure models are not merely fitting noise but capture the underlying system dynamics robustly, especially for complex biological systems like microbial communities.

### Overall assessment of the workflow

The initial phase of our workflow uses structural identifiability analysis (SIA) to confirm whether model parameters can be uniquely determined from a given experimental design. The analysis often reveals that while models might be identifiable with perfect data, they become unidentifiable with realistic, limited measurements, such as partially observed scenarios. Without performing SIA, a modeler risks wasting enormous time and computational resources trying to fit a model whose parameters are fundamentally unknowable from the combination of inputs and outputs in the experimental design. They would be attempting an impossible task, leading to non-unique solutions where countless different parameter sets produce the exact same model output. This makes it impossible to draw reliable conclusions, turning the modeling effort into a futile exercise and completely undermining any claim of mechanistic insight. This workflow helps avoid such a dead-end by identifying structural limitations *before* the intensive estimation process begins.

Following this, the parameter estimation phase of our workflow addresses the dual challenges of finding the best-fit parameters and ensuring those parameters are themselves reliable. The results show that standard, local optimization methods (widely used) often fail for complex models, whereas a global optimizer is far more robust. Critically, even a model that perfectly fits the data can suffer from poor practical identifiability, where parameters have massive uncertainty. By using inadequate optimization tools, a modeler might wrongly conclude that their model is flawed, when in fact the tool was simply not powerful enough to find the global solution. More dangerously, by stopping at a good fit without assessing practical identifiability, a modeler can fall into a major trap, leading to false scientific claims. The model may look perfect, but the estimated parameters could be meaningless, leading to incorrect biological interpretations. For example, reporting a competitive interaction that is actually symbiotic. This mischaracterization of the system’s dynamics can misdirect future experiments and lead to a fundamental misunderstanding of the biological system. Our workflow helps to prevents such erroneous conclusions by demanding that the parameter estimates themselves be trustworthy.

The final phase uses cross-validation to assess a model’s ability to predict outcomes under new conditions, which is the ultimate test of its validity. The analysis confirms that overfitting (capturing noise rather than the true underlying dynamics) is a severe risk, especially in gLV models. Neglecting rigorous validation creates a model that may perfectly describe the training data but is useless for its primary purpose: prediction. When faced with new scenarios, the overfitted model will fail spectacularly, producing inaccurate and unreliable forecasts. This renders the model untrustworthy for any practical application, such as designing a stable microbial consortium or predicting the effect of a treatment. Our workflow helps to avoid this critical failure by ensuring the model is not just a descriptive snapshot of old data, but a robust tool capable of generating reliable predictions about the future.

In this context, it is important to note that several studies have highlighted fundamental limitations of generalized Lotka-Volterra (gLV) models in microbial community dynamics. Momeni et al. [58] critique the universality assumption of pairwise Lotka-Volterra models, showing through mechanistic references that diverse chemical-mediated interactions demand tailored mathematical forms, as mismatched models yield erroneous predictions on dominance or coexistence. Similarly, Hart et al. [59] found that parameters from standard batch cultures failed to predict growth in a synthetic cooperative yeast community due to environment-dependent phenotypes like metabolite rates. Picot et al. [14] further argue that gLV’s assumption of dynamic equilibrium clashes with batch cultures’ static resource-exhausted endpoints, fostering overfitting to early growth phases and unreliable long-term predictions with setup-sensitive parameters.

While these studies point to fundamental problems with Lotka-Volterra models, our work identifies additional, critical challenges. We reveal that these models might also suffer from structural and practical non-identifiability, local optima (underfitting), and numerical instabilities. Our research therefore complements previous findings and serves as a clear warning: the popular gLV framework has numerous pitfalls, and its application to dynamic microbial modeling requires significant caution.

## Discussion

Modeling the complex dynamics of microbial communities using nonlinear ODEs presents significant challenges, often hindering the development of reliable and predictive models. In this study, we proposed and evaluated an integrated computational workflow designed to systematically address four critical pitfalls inherent in the identification process: structural and practical identifiability issues, numerical instabilities leading to finite-time blow-up during estimation, convergence to suboptimal solutions (underfitting), and fitting experimental noise rather than the underlying signal (overfitting). Our results across a range of case studies, from simple gLV models to complex representations of gut microbiomes and synthetic systems (Table 1), demonstrate the prevalence of these issues and the necessity of a structured approach to mitigate them.

Our approach is motivated by a unique set of challenges that distinguish these models of microbial communities from other typical systems biology models (such as e.g. metabolic or signaling networks). Beyond being high-dimensional and nonlinear, they often feature dense interaction structures, network symmetries that worsen identifiability, and numerical instabilities like solution blow-up, very especially in gLV formulations. As illustrated with our results, the severity of these combined issues makes the estimation problem particularly hard, thus demanding the tailored methodology we have developed. We propose an integrated workflow to address four modeling pitfalls simultaneously. This research introduces a comprehensive and systematic methodology that concurrently tackles identifiability issues, model instability (blow-up), underfitting, and overfitting. While these challenges are known individually, this paper’s contribution is combining their analysis and mitigation into a single, structured pipeline. This integrated approach is specifically tailored for the complexities of dynamic microbial community models.

The paper demonstrates the effectiveness of its proposed workflow by applying it to a variety of canonical models representing microbial communities, ranging from simple two-species systems to a more complex twelve-species gut microbiome model. This rigorous testing across different model structures and complexities showcases the versatility and practical utility of the approach. The study also provides the software implementation, enhancing reproducibility and accessibility for other researchers. While the most complex case study (a 12-species generalized Lotka–Volterra model) still represents a substantial simplification of real microbial ecosystems such as the human gut microbiome, it nonetheless provides a valuable intermediate step between toy models and full-scale community simulations. In fact, in the context of dynamic modeling of microbial communities, models of this size are among the most complex that have been systematically analyzed, particularly with respect to structural and practical identifiability. Most prior studies have focused on smaller systems due to the computational and analytical challenges involved. Our results show that even in these moderately complex and controlled settings, significant identifiability issues can arise. This suggests that such challenges are likely to be even more pronounced in more realistic, higher-dimensional microbiome models. By focusing on these tractable yet nontrivial systems, our study highlights methodological pitfalls and limitations that must be addressed before scaling up to more realistic, data-driven applications.

**Fig 4:**
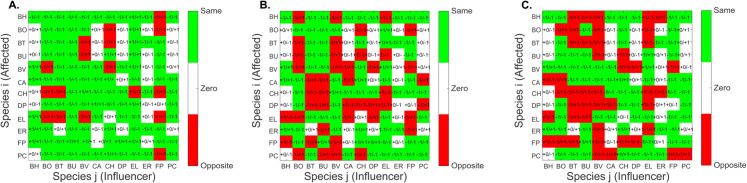
Case study *MGLV*: Sign agreement between estimated and nominal values of the interaction coefficient $\beta_{ii}$. The figures correspond to successful calibrations to data with 0, 5 and 10% noise. Details provided in S1 Text (Section 7.10.4).

Identifiability is a cornerstone of reliable parameter estimation. Our workflow begins with structural identifiability analysis (SIA) in Phase 1, which, as shown in our results (Table 2), effectively flags theoretical limitations based on model structure and observation schemes before estimation commences. This step can guide experimental design by highlighting necessary measurements (e.g., demonstrating insufficiency of total biomass measurement in GLV2). However, even structurally identifiable models often suffer from practical non-identifiability, especially with increasing complexity and noise, as revealed by our practical identifiability analysis (PIA) in Phase 2. This was manifested through wide confidence intervals, parameter correlations, and, critically, incorrect sign estimations for interaction parameters (e.g., Fig 4), leading to mechanistically unsound models despite potentially good data fits. Addressing both SIA and PIA is crucial for obtaining meaningful parameter estimates.

The parameter estimation phase itself is fraught with difficulties arising from non-convex optimization landscapes exacerbated by potential model instabilities. Our findings highlight the limitations of standard multistart local optimization methods, which frequently failed due to encountering finite-time blow-up regions or converged to poor local optima (underfitting), as exemplified by the GLV3 results (Fig 2). The use of robust global optimization techniques, such as the enhanced Scatter Search (eSS) employed in Phase 2, proved significantly more effective in navigating these complex landscapes, reducing the incidence of underfitting and managing numerical integration failures caused by blow-ups, thereby increasing the likelihood of finding globally competitive solutions, even for challenging high-dimensional models like MGLV.

Achieving a good fit to the training data is insufficient proof of a model’s validity. Overfitting, where the model captures noise, poses a major threat to predictive power. Our results clearly demonstrated instances where models fit the training data well but failed dramatically when tested on unseen data via cross-validation (Phase 3, Fig 3). This underscores the indispensable role of cross-validation in assessing generalization capability. While cross-validation using additional datasets is preferred, residual analysis (e.g., Q-Q plots, see S1 Text, Section 7.6.4) offers a potential alternative check when obtaining such data is prohibitive. Detecting and avoiding overfitting is paramount for developing models that provide reliable predictions beyond the calibration conditions.

Crucially, our results demonstrate that the four pitfalls (identifiability, blow-up, underfitting, and overfitting) are often interconnected and can exacerbate one another. For instance, poor identifiability increases the risk of overfitting or converging to local minima. Blow-up dynamics can completely derail the search for meaningful parameters. Failing to address these intertwined problems systematically leads to significant modeling artifacts, as seen across our case studies. For example, in GLV models such artifacts manifest as unreliable parameters, often with incorrect signs that misrepresent biological mechanisms. Consequently, these models predict poorly under new conditions and can lead to flawed biological interpretations. This adds to the existing evidence [14,58,59] that gLV models, despite their popularity, are fraught with potential pitfalls and should be used with extreme caution in microbial ecology.

Therefore, considerable care and rigorous validation, extending beyond simple goodness-of-fit checks, are essential when developing and applying dynamic models of microbial ecosystems. The integrated, multi-phase workflow presented here provides a structured methodology. It allows researchers to diagnose and mitigate these common pitfalls sequentially. This approach fosters the development of more robust, mechanistically plausible, and predictively powerful models, vital for advancing our understanding of microbial communities.

## Supporting information

S1 Text Additional information supplementing this manuscript. (PDF)
